# Communication of Pulmonary Function Test Results: A Survey of Patient’s Preferences

**DOI:** 10.1371/journal.pone.0126617

**Published:** 2015-05-07

**Authors:** Debbie Zagami, Jessica Hockenhull, Alanna Bodger, Krishna Bajee Sriram

**Affiliations:** 1 Department of Respiratory Medicine, Gold Coast University Hospital, 1 Hospital Boulevard, Southport, Queensland, Australia; 2 School of Medicine, Parklands Drive, Griffith University, Southport, Queensland Australia; Postgraduate Institute of Medical Education and Research, INDIA

## Abstract

**Introduction:**

Physician-patient communication in patients suffering from common chronic respiratory disease should encompass discussion about pulmonary function test (PFT) results, diagnosis, disease education, smoking cessation and optimising inhaler technique. Previous studies have identified that patients with chronic respiratory disease/s often express dissatisfaction about physician communication. Currently there is a paucity of data regarding patient awareness of their PFT results (among those who have undergone PFTs previously) or patient preferences about PFT result communication.

**Methods:**

We undertook a three-month prospective study on outpatients referred to two Pulmonary Function Laboratories. If subjects had undergone PFTs previously, the awareness of their previous test results was evaluated. All subjects were asked about their preferences for PFT result communication. Subjects were determined to have chronic respiratory disease based on their past medical history.

**Results:**

300 subjects (50% male) with a median age (±SD) of 65 (±14) years participated in the study. 99% of the study participants stated that they were at least moderately interested in knowing their PFT results. 72% (217/300) of the subjects had undergone at least one PFT in the past, 48% of whom stated they had not been made aware of their results. Fewer subjects with chronic respiratory disease preferred that only a doctor discuss their PFT results with them (28% vs. 41%, p = 0.021).

**Conclusion:**

Our study demonstrates that while almost all subjects want to be informed of their PFT results, this does not occur in a large number of patients. Many subjects are agreeable for their PFT results to be communicated to them by clinicians other than doctors. Further research is required to develop an efficient method of conveying PFT results that will improve patient satisfaction and health outcomes.

## Introduction

Chronic respiratory diseases such as asthma, chronic obstructive pulmonary disease (COPD) and interstitial lung diseases, are associated with considerable morbidity and mortality in both developed and developing nations worldwide [1]. The diagnosis of chronic respiratory diseases typically consists of a comprehensive assessment of patients’ symptoms, pulmonary function tests (PFTs), radiological imaging, health and functional status and quality of life evaluations [2–4]. Once the diagnosis of chronic respiratory disease is established, adequate patient education is very important in achieving improvements in important health outcomes [5, 6]. The ideal physician-patient communication in patients with the common respiratory diseases will most likely be multi-faceted consisting of (but not restricted to) communicating diagnosis, test results, disease education and management plan/s. It is now well recognised that effective physician-patient communication can positively influence a patient's adherence to treatment [7].

Communicating PFT results to patients with chronic respiratory diseases is an integral component of a successful patient education and self-management strategy. Research demonstrates that one of the unmet needs for patients and caregivers is better physician-patient communication relating to interpretation of diagnostic test results [8–14]. One small study assessed physician-patient communication in COPD outpatient visits and found that spirometry test results were only discussed in 69% of instances [14]. This is concerning since improved patient awareness of their own PFT results may assist them in relating the objective measurements to their symptoms and connect with their diagnosis and treatment plan. Furthermore, some studies have reported that communicating spirometry results to smokers resulted in increased smoking cessation rates [15–20].

Indeed, it has been our experience the physician communication of PFT results to patients is often an overlooked aspect of chronic respiratory disease management. To the best of our knowledge, patient awareness of their PFT results or their preferences for the communication of PFT results has not been specifically evaluated previously. This is surprising since PFTs are being performed in increasing numbers and in the majority of instances PFTs are being performed in patients with chronic respiratory disease to follow disease progression or response to treatment [21].

The aims of this study were to document patient awareness of previous PFT results, their interest and their preference for the communication of PFT results. It is hoped the results of our study would provide us with much needed information that can be used to develop a protocol for the appropriate and adequate communication of PFT results to patients.

## Methods

### Subjects

Consecutive adult (age ≥18 years old) outpatients who were booked to undergo PFTs (spirometry, bronchodilator reversibility assessment, diffusion capacity measurement, lung volume measurement and fractional exhaled nitric oxide testing) were considered for the survey. The study was undertaken in the Pulmonary Function Laboratories of the Gold Coast Hospital and Health Service (GCHHS) between May and August 2014. The GCHHS comprises two regional hospitals, the Gold Coast University Hospital (570-bed) and Robina Hospital (300-bed) both of which operate pulmonary function Laboratories. The laboratories provide PFT services to only GCHHS outpatients and in-patients. After completion of their PFTs, subjects were invited to participate in the study. All participating subjects provided written informed consent. Review of the medical records was used determine if a subject suffered from a chronic respiratory disease (COPD, Asthma, Bronchiectasis, Interstitial lung disease). The study was approved by the GCHHS Human Research Ethics Committee (HREC 13/QGC/188).

### Questionnaire

Basic demographic information (including age, smoking status and sex) and educational attainment was collected from consenting subjects. Subjects were given the questionnaire by one of three respiratory scientists (DZ, JH, AB). The questionnaire was designed to evaluate subjects' understanding and awareness of their underlying pulmonary disease (if any), self-perception of the severity of their underlying lung disease, awareness of previous PFT results (if applicable); recall of whether the previous PFTs were explained to patient; interest in knowing the results of their current PFT results; patient preference only a doctor should discuss PFT results with them; difficulty in performing PFTs and willingness to perform PFTs again. Responses were tailored to a multiple-choice format to aid completion.

The PFT report was reviewed to collate the following information: reason for referral to PFT laboratory; physician diagnosis (i.e. was the patient known to have a chronic respiratory disease); specialisation of the requesting physician (i.e. pulmonary specialist versus non-pulmonary specialist); physician report of the patients PFT results (pattern and severity of abnormality).

### Statistical Analysis

All statistical analyses were performed using the statistical software Statistical Package for the Social Sciences (SPSS, Version 20, SPSS Inc., Chicago, USA). Descriptive statistics were summarised for demographic and social factors and reported as means and standard deviations. Kolmogorov-Smirnov and Shapiro-Wilk tests were used to assess normality of the data. Normally distributed data was analysed for differences between individual groups using the Student's *t*-test, nonparametric data was analysed for differences between groups using the Mann-Whitney U test and p-values less than 0.05 were considered significant.

## Results

Among the initial set of 348 eligible subjects, 21 declined to participate in the study, 11 had difficulty comprehending the questionnaire, 16 patients could not complete the questionnaire as they had to leave early for another medical appointment. The data for the remaining 300 subjects was used for the study (Table 1). 40% of subjects had attained at least a tertiary education. 56% of the study participants stated they were former smokers and 19% stated that they were current smokers. Spirometry testing was performed in 299 (99%) subjects, lung volume measurement in 239 (80%) subjects and diffusion capacity in 263 (88%) subjects.

**Table 1:** Characteristics of Respondents.

Characteristics	Subjects with Known Chronic Respiratory Disease (N = 131)	Subjects without Known Chronic Respiratory Disease (N = 169)	p-value
**Sex**			
Male	66 (50.4%)	85 (49.7%)	1.000
Female	65 (49.6%)	85 (50.3%)	
**Age, years**			
Mean (SD)	63 (15)	63 (14)	0.627
Range	18–89	21–89	
**Highest level of schooling**			
Primary	28 (21.4%)	36 (21.3%)	0.698
Secondary	48 (36.6%)	68 (40.2%)	
Tertiary	55 (42%)	65 (38.5%)	
**Smoking status**			
Never smoked	30 (22.9%)	46 (27.2%)	0.959
Former smokers	79 (60.3%)	88 (52.1%)	
Current smokers	22 (16.8%)	35 (20.7%)	
**Clinician requesting current PFT**			
Respiratory physicians	99 (75.6%)	103 (61%)	**0.009**
Other hospital clinicians	32 (24.4%)	66 (39%)	
**PFTs requested**			
Spirometry	130 (99%)	169 (100%	1.000
Bronchodilator reversibility assessment	45 (34%)	47 (28%)	0.256
Lung volumes	104 (79%)	135 (80%)	1.000
Diffusion capacity	109 (83%)	154 (91%)	0.051
**Major reasons for PFT referral**			
	Known respiratory disease-COPD: 58 (44%)	Assess lung function in Connective Tissue Disease (CTD): 1 (0.6%)	N/A
	Known respiratory disease-Asthma: 18 (13.7%)	Assess lung function in other organ disease: 1 (0.6%)	
	Known respiratory disease-Bronchiectasis: 19 (14.5%)	Investigation of specific disease-Asthma: 17 (10.1%)	
	Known respiratory disease-Interstitial Lung Disease (ILD): 27 (20.6%)	Investigation of specific disease-COPD: 23 (13.6%)	
	Known respiratory disease-Other: 5 (3.8%)	Investigation of specific disease-ILD: 16 (9.5%)	
	Pre-operative assessment—Lung Cancer: 2 (1.5%)	Investigation of specific symptoms-dyspnoea 44 (26%)	
	Pre-operative assessment-Other: 2 (1.5%)	Investigation of specific symptom-cough: 6 (3.6%)	
		Possible induced lung disease-Occupational: 4 (2.4%)	
		Pre-operative assessment—Lung Cancer: 25 (14.8%)	
		Pre-operative assessment-Other: 32 (18.9%)	

Among the study subjects, the interest to know the results of the PFTs was almost universal (299/300). 217 subjects stated that they had performed spirometry and/or other PFTs in the past. Among these, 48% stated that previous PFT results had not been discussed with them. Of those who stated that their PFT results has been discussed with them, there was a strong correlation between the subjects’ self-perception of the severity of their lung disease and the physician’s assessment of the PFT results (Pearson correlation coefficient of 0.495, p<0.001). However, among subjects who stated that their PFT results had not been discussed with them, there was no correlation between their self-perception of the severity of their lung disease and the physicians’s assessment of the PFT results (Pearson correlation coefficient of 0.169, p = 0.21).

One hundred and thirty one subjects had been previously diagnosed with chronic respiratory disease and the remaining 169 subjects had no known chronic respiratory disease. There was no sex difference among the respondents. Respiratory physicians were the requesting clinicians more often in subjects with chronic respiratory disease compared to subjects without known chronic respiratory disease (74.8% vs. 26.6%, p<0.001). The most common reason for referral for PFT among subjects with chronic respiratory disease was COPD (44%), while among subjects without known chronic respiratory disease, it was for pre-operative evaluation for non-lung cancer related procedures (18.9%). Not surprisingly, subjects with known chronic respiratory disease were more likely to report that they suffered from a lung disease (74.8% vs. 26.6%, p<0.001) subjects without chronic respiratory disease were more likely to be unaware of the severity of their lung disease (85% vs. 53%, p<0.001).

Subjects with chronic respiratory disease had undergone PFTs previously more often than subjects without known chronic respiratory disease (84% vs. 63%, p<0.001) (Table 2). Fewer subjects with chronic respiratory disease wanted a doctor to discuss their PFT results with them compared to subjects without chronic respiratory disease (28.2% vs. 41.4%. p = 0.021). While subjects with chronic respiratory disease reported that they found it difficult to perform PFTs compared to subjects without chronic respiratory disease (26.7% vs. 13.6%, p = 0.005), almost all subjects were willing to undergo repeat PFTs in the future if required (99.2% vs. 98.8%, p = 1.000).

**Table 2:** Respondents Beliefs Relating to Pulmonary Function Tests.

	Subjects with Known Chronic respiratory disease (N = 131)	Subjects without Known Chronic respiratory disease (N = 169)	p-value
**I suffer from a lung disease**	98 (74.8)	45 (26.6%)	**<0.001**
**I don't know the severity of my lung disease**	69 (53%)	143 (85%)	**<0.001**
**I have had PFTs before**	110 (84%)	107 (63%)	**<0.001**
My previous PFT results were explained to me*	57 (52%)	56 (52%)	1.000
**I would like to know the results of my PFTs**	128 (97.7%)	169 (100%)	1.000
**I only want a doctor to explain the PFT results to me**	37 (28.2%)	70 (41.4%)	**0.021**
**I found it difficult to perform the PFTs**	35 (26.7%)	23 (13.6%)	**0.005**
**I am willing to have a PFT again**	130 (99.2%)	167 (98.8%)	1.000

* Only subjects who had PFTs in the past were included

## Discussion

In patients with chronic respiratory diseases, such as COPD, asthma and interstitial lung disease, physician-patient communication may need to encompass PFT results, smoking cessation, medications, lifestyle changes and if appropriate advanced care planning as well [2–4]. However, it has been recognised that patients are often are dissatisfied with physician communication surrounding chronic respiratory disease diagnosis and management plans [14, 22]. One area of communication that has been highlighted that could readily be improved is physician patient communication of PFT results [14]. In the wider context of diagnostic tests, it is recognised that in some instances only one-third of patients are informed about abnormal test results and the majority of patients with normal results are not informed about their results [11]. On the contrary more than 90% of patients express a desire to be informed of both normal and abnormal results [23]. The lack of communication of test results can result in patient harm and has been found to contribute to about 45% of malpractice claims [24]. While the importance of patient communication about test results is well recognised in the literature, it is also noted that the issue of communicating test results is complex. Not surprisingly, there are no formal guidelines directing or assisting clinicians in the most appropriate method of communicating test results.

For patients with chronic respiratory disease, being aware of their PFT results may influence their health behaviour such as smoking cessation. Cigarette smoking is by far the most important risk factor for COPD, and smoking cessation is the single most effective way to reduce the risk of developing COPD and to affect the clinical outcome in all stages of the disease [3]. Discussing abnormal test results with smokers has been suggested to be a ‘‘teachable moment” that may increase motivation to quit smoking, albeit the evidence to support such an approach is weak [16, 25–27]. In our study, 52% of subjects stated that they had not been informed about their PFT results. Furthermore 57 (19%) subjects stated that they were current smokers. While it is still unresolved in the literature that providing smokers with their PFT results, particularly if they are abnormal may increase their smoking abstinence rates [15, 18], it will nevertheless provide them with more information about their health status. Discussing PFT results may also prompt the physician to initiate and prioritize smoking cessation during the consultation.

The process of physician-patient communication of disease diagnosis, PFT results and management plans in our institution is informal with no protocol in place and we believe such a practice is common in other institutions as well. Interestingly, Litchfield also reported that most practices also lack any systematic protocol for communicating the results of even commonly performed laboratory tests to patients [24]. A lack of a systematic approach to patient evaluation and communication has been found to be associated with increased patient dissatisfaction, particularly for COPD patients [28]. There is a need for the development of newer models of care for the management of patients with chronic respiratory disease, where there is sufficient time and resources to canvass patients health beliefs, provide adequate information and implement effective management and treatment plans.

One strategy that has been suggested is to strengthen the role of a nurse practitioner to complement the role of the physician in the management of patients with chronic respiratory diseases [29]. The scope of the nurse practitioner within a multi-disciplinary team could include diagnosis, discussing PFT results, education and patient monitoring [29]. In our study, 64% of study subjects stated that they were willing to discuss their PFT results with a health care professional other than a doctor. This implies that there is scope for non-physician members of a multi-disciplinary disease management team to be more proactively involved in the diagnosis and management of patients with chronic respiratory disease. The feasibility of such an approach requires further study.

While it was not one of our study objectives, it was interesting to note that almost 80% of subjects performed complete lung volume measurement assessments. A recent study by Pretto et al, also identified this practice of routinely assessing complex lung function measurements [21]. This is concerning because the clinical utility and significance of lung volume measurements over and above spirometry, has not been categorically demonstrated. Also in our study we found that a little over a quarter of subjects with chronic respiratory disease found it difficult to perform PFTs. Physicians may need education and support regarding the appropriateness of routinely requesting complete lung volume measurements with little consideration to the costs involved both to the patients and health service in general.

Strengths of this study include the good response rate and ability to assess a previously unanswered question about patient preferences for PFT test result communication. Limitations of the study include the lack of generalizability since the survey was only conducted at two PFT laboratories. Also, the study did not explore physician preferences about PFT result communication. It was also beyond the scope of the study to evaluate the clinical impact of the PFT result on patient management. It is possible that PFT results indeed may have been communicated to a much higher proportion of study subjects and the subjects simply do not remember the discussion. It has been previously noted that patients forget 40–80% of medical information provided by health professionals [30]. Nevertheless, we found that subjects who stated that their PFT results were communicated to them, had a moderately strong correlation between self-perception of pulmonary disease and their actual PFT results. Our results suggest that there is at least moderate effectiveness of PFT result communication on patients' understanding of their pulmonary health.

Future research should assess the ability of allied health professionals to appropriately communicate with patients regarding their test results, disease diagnosis and management patients in order to improve patient satisfaction. This may provide an opportunity for improvements in patient satisfaction and health care delivery for patients with both acute and chronic diseases.
